# Tinea Versicolor in a Three-Month Infant: A Case Report and Literature Review

**DOI:** 10.7759/cureus.40763

**Published:** 2023-06-21

**Authors:** Marwah K Almalki, Ziyad M Alruwaili, Norah S Alhammad, Toleen M Alawadi, Mazen S Dajam

**Affiliations:** 1 Faculty of Medicine, Al-Rayan Medical College, Al-Madinah Al-Munawwarah, SAU; 2 Internal Medicine, Jouf University, Jouf, SAU; 3 Dermatology, King Fahad Armed Forces Hospital, Jeddah, SAU

**Keywords:** hypopigmented macules, malassezia, infant, pityriasis versicolor, tinea versicolor

## Abstract

Tinea versicolor (TV) is a superficial fungal disease caused by Malassezia furfur, most commonly affecting adolescents and adults. TV is uncommon among newborns, particularly those aged under one year. Poor hygiene and perspiration, immunosuppression, the use of oils and greasy lotions, hyperhidrosis, and corticosteroids may all contribute to the appearance of the condition. It is clinically distinguished by hypopigmentation or hyperpigmentation. Most often, it occurs over the trunk. Treatment for TV usually begins with the use of a topical antifungal. This case report presents a rare TV in a three-month-old boy who exhibited multiple hypopigmented macules on his trunk without pruritus. Examination of the wood lamp showed bright yellow fluorescent lesions. The potassium hydroxide (KOH) preparation revealed yeast and short mycelial forms, confirming the diagnosis of TV. The baby was given a clotrimazole solution for topical use twice a day. KOH preparation was negative, and the lesions had improved at the time of the two-month follow-up. This case highlights the importance of considering TV as a potential diagnosis in infants with atypical skin manifestations, although it is more commonly seen in older individuals.

## Introduction

Tinea versicolor (TV) is a superficial fungal infection of the skin caused by Malassezia furfur, which most commonly occurs in adolescents and adults. However, it is believed that TV can happen to younger children in tropical areas [1]. Several factors, including poor hygiene and sweating, immunosuppression, diabetes mellitus, the use of oils and oily creams, hyperhidrosis, and corticosteroids, have been established to contribute to the appearance of the disease [2]. Hypopigmented or hyperpigmented macule patches clinically distinguish them. Most often, it occurs over the trunk. In infants, the manifestation of TV is relatively rare due to the immaturity of the sebaceous glands and the lower production of sebum, which serves as a source of Malassezia [3]. However, infant and newborn cases have been reported, with clinical presentations that differ from those observed in adolescents and adults [4]. TV in infants tends to be more inflammatory, and lesions usually spread rapidly and are more challenging to treat [5]. The use of a topical antifungal is the initial therapy for the treatment of TV [6].

## Case presentation

**Figure 1:**
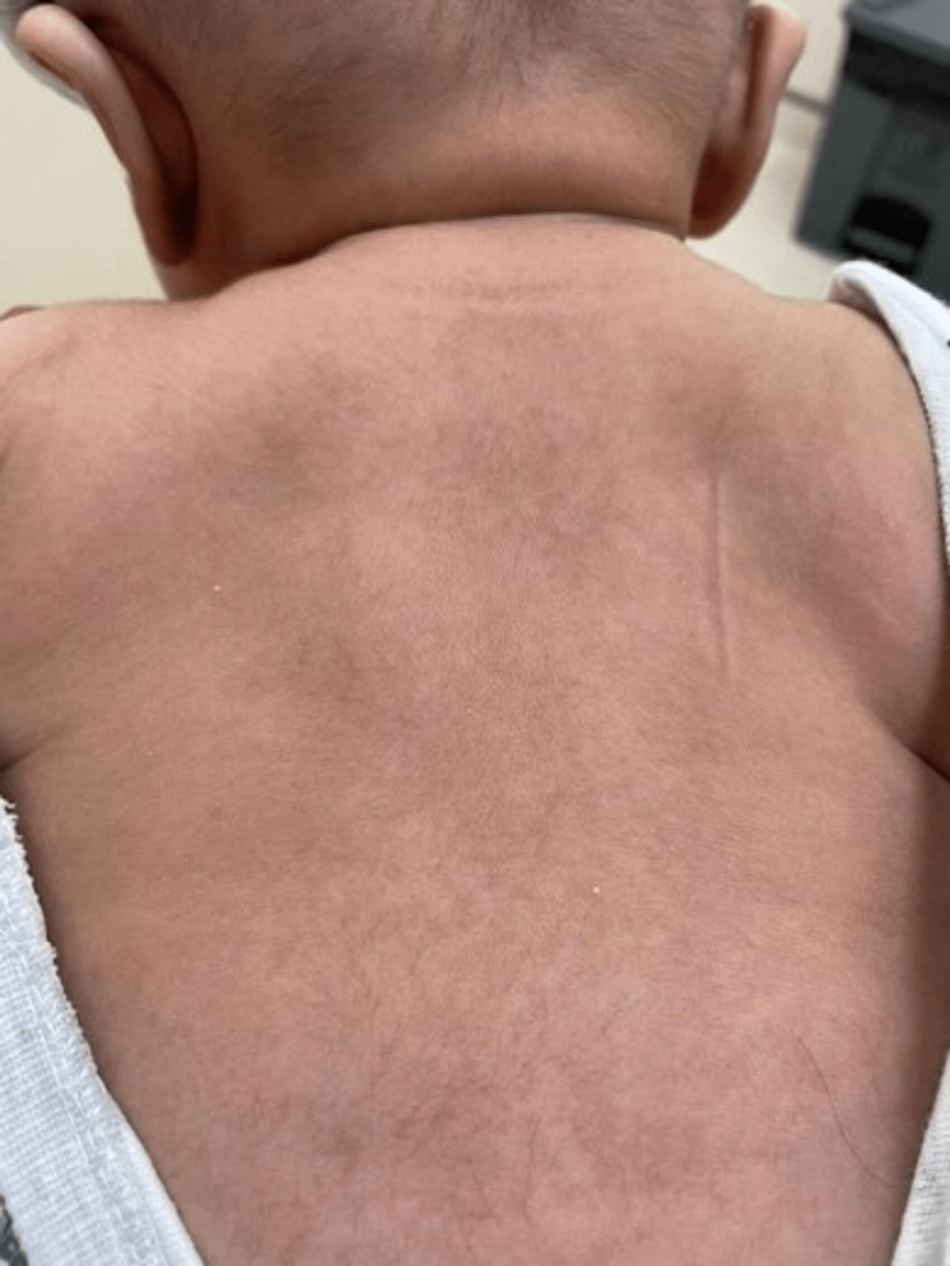
Multiple hypopigmented macules covered with fine scales over the back.

**Figure 2:**
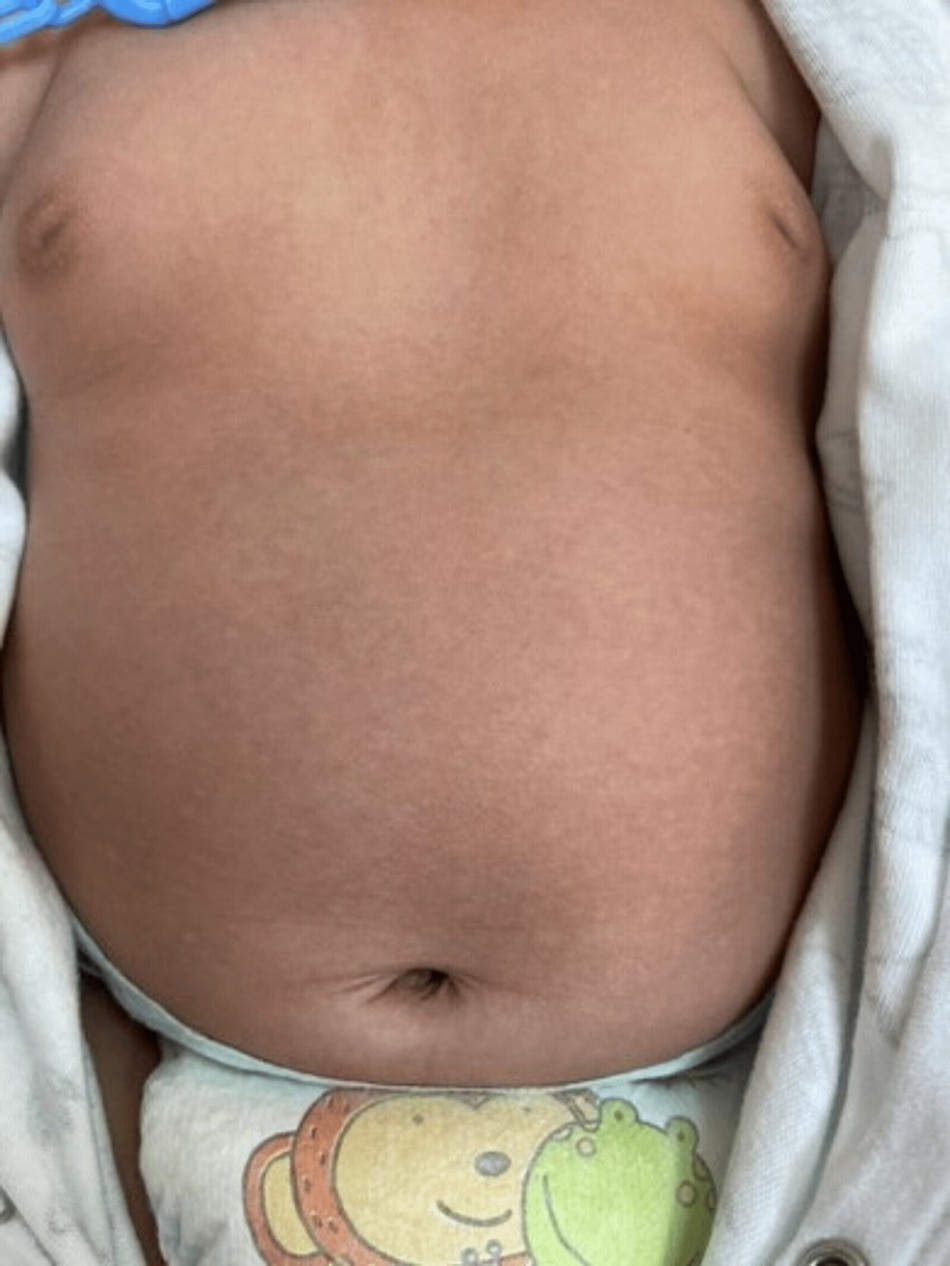
Multiple hypopigmented macules covered with fine scales over the chest.

**Figure 3:**
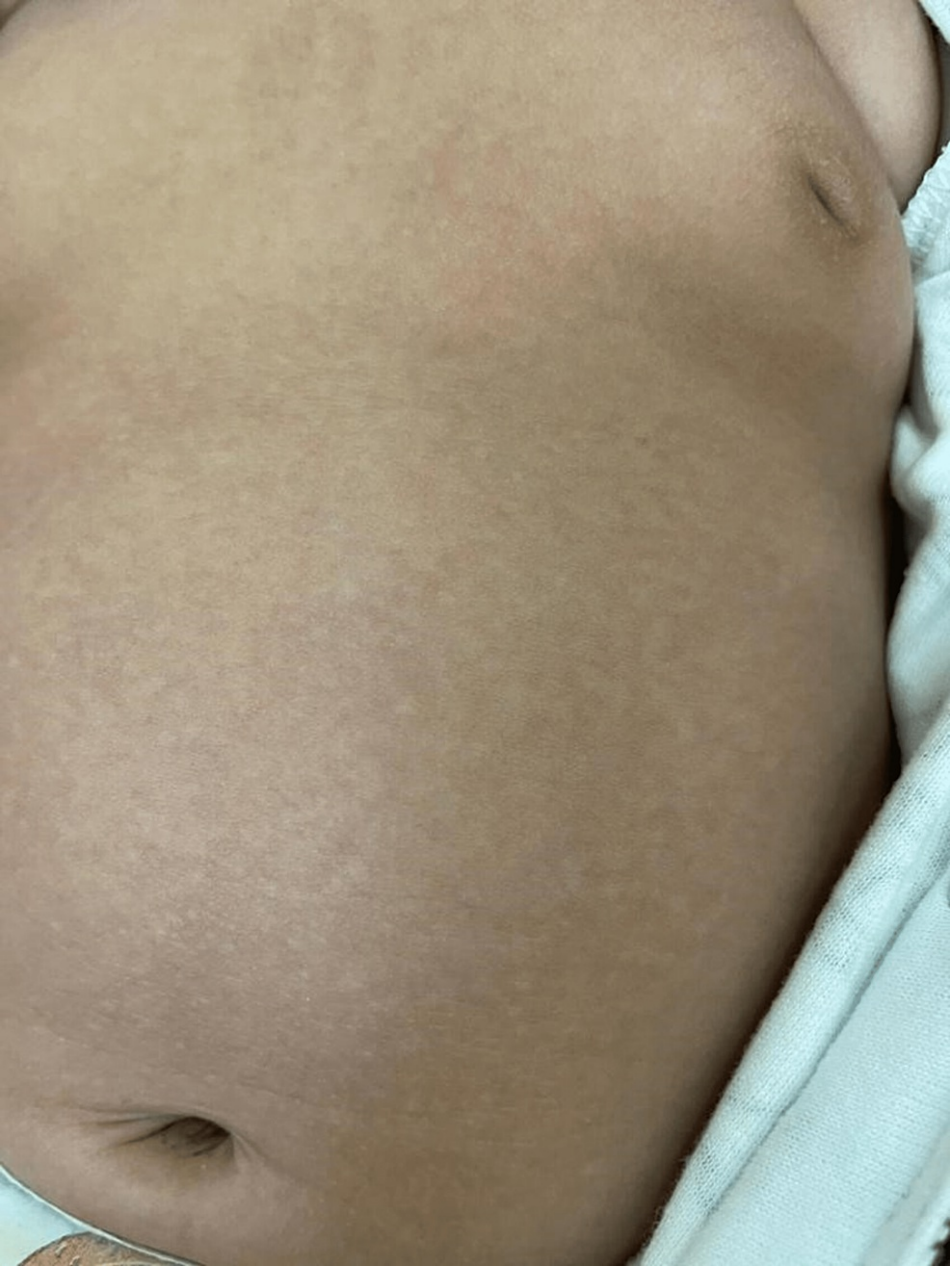
Multiple hypopigmented macules covered with fine scales over the abdomen.

A three-month-old boy was presented to our dermatology clinic with multiple hypopigmented macules on his trunk for two weeks without pruritus. He was delivered by normal vaginal delivery at 39 weeks gestational age and weighed 3.6 kg. Due to physiological jaundice, he was kept in the nursery for three days. Neither steroids nor antibiotics were used. He was not using a central venous catheter or total parenteral nutrition (TPN). On physical examination, multiple hypopigmented macules were covered with fine scales on the back (Figure 1), chest (Figure 2), and abdomen (Figure 3).

Examination with a wood lamp showed bright-yellow fluorescent lesions. A potassium hydroxide (KOH) examination revealed yeast and short mycelial forms that resemble *ziti and meatballs*, supporting the diagnosis of TV. The mother was advised to apply a 1% clotrimazole solution twice daily, and follow-up after two months showed negative KOH preparation and improvement of the lesions.

 

## Discussion

TV rarely occurs in childhood. However, it is believed that TV can happen to younger children in tropical areas [1]. Several factors, including poor hygiene and sweating, immunosuppression, diabetes mellitus, the use of oils and oily creams, hyperhidrosis, and corticosteroids, have been established to contribute to the appearance of the disease [2] and clinically distinguished by hypopigmentation or hyperpigmentation, most commonly on areas of skin that are sebum-rich, such as the upper arms, trunk, neck, and shoulders. Although facial involvement is rare in adults, it is common in children and may be the only site affected.

TV should be diagnosed clinically, and a KOH preparation test can be used to identify clusters and many short, stubby hyphae [5]. A topical antifungal for four to six weeks is the initial therapy for TV treatment.

Topical antifungal therapy has a lower cost, fewer drug-drug interactions, a higher efficacy, a safer profile, and greater compliance when treating TV in comparison to oral antifungal, laser, and photodynamic therapies, which are reserved for refractory and recurrent cases [2].

To our knowledge, there are only 11 case reports of TV in infants [1,2,7-9]. Here, we report cases of hypopigmented TV and review the literature.

In 1984, the first TV case was reported in childhood [10], and subsequently, more cases have been reported since then [1,2,7-9]. Eight of the 11 cases were males; two had a positive family history of TV. Regarding the medical history, one case is known to have atopic dermatitis, and another patient was born prematurely with low birth weight (LBW) on TPN and required admission to the intensive care unit (ICU) and antibiotic therapy. Although others do not have a significant medical history, almost six cases had negative KOH tests, and most patients had favorable responses to topical antifungal treatments (Table 1).

**Table 1:** Reported cases of infantile pityriasis versicolor (under the age of one year). LBW, low birth weight; TPN, total parenteral nutrition; ICU, intensive care unit; NA, not available; NVD, normal vaginal delivery

Author (Year) Reference	Sex/Age	Region	Physical exam	Location	Family history	Past medical history	Delivery	Treatment
Congly (1984) [10]	Male/3 months	Saskatchewan	Erythematous scaly macules and patches	Dorsal aspect of the upper arm, shoulders, and upper back	Negative	Negative	NA	Clotrimazole 1% solution
Di Silverio et al. (1995) [7]	Male/2 months	Italy	Hyper- and hypopigmented scaly macules	Cervical, scalp, face, and upper chest	Negative	Negative	NVD	Econazole 1% lotion
Nanda et al. (1998) [1]	Male/3 weeks	India	Several hypopigmented macules	Forehead	Negative	Negative	NVD	Clotrimazole 1% solution
	Male/4 months	India	Hypopigmented scaly lesion	Neck, upper trunk, arms, and face	Positive	Negative	NA	Clotrimazole 1% solution
	Male/5 months	India	Light-brown, scaly macules	Neck	Negative	Atopic dermatitis	NA	Clotrimazole 1% solution
	Male/4 weeks	India	Hypopigmented scaly macules	Forehead	Negative	NA	NVD	Clotrimazole 1% solution
	Female/5 weeks	India	Hypopigmented scaly macules	Face and forehead	Negative	NA	NA	Clotrimazole 1% solution
Jubert et al. (2015) [8]	Male/3 weeks	Spain	Hypopigmented macules and patches	Upper trunk, face, and neck	Negative	Premature, LBW, TPN, ICU admission, and antibiotic therapy	NA	Intravenous fluconazole
Said et al. (2010) [9]	Male/3 months	Tunisia	Hypopigmented macules	Cervical and chest	Positive	Negative	NA	Topical antifungal
Abdollahimajd et al. (2019) [2]	Female/8 months	Iran	Hypopigmented macules	Lateral face, neck, upper back, and chest	Negative	Negative	NVD	Topical antifungal
	Female/4 months	Iran	Hypopigmented macules	On the frontal area of the face	Negative	Negative	NVD	Topical antifungal
Present case	Male/3 months	Pakistan	Hypopigmented scaly macules	Trunk	Negative	Negative	NVD	Clotrimazole 1% solution

## Conclusions

TV is a superficial fungal infection that often affects the back and chest in young adults. It is rare in infants, especially those under one year of age, and when it occurs, affected children usually present atypical symptoms. Despite the atypical presentation in this age group, the diagnosis was confirmed through Wood's lamp examination and KOH testing. Successful treatment with a 1% clotrimazole solution led to a significant improvement in the patient's condition. This case highlights the importance for dermatologists and healthcare professionals to consider TV as a potential diagnosis in infants with unusual skin manifestations, particularly in tropical areas where the condition may be more prevalent. Early diagnosis and appropriate antifungal treatment can lead to favorable outcomes and prevent potential complications.

## References

[1] Nanda A, Kaur S, Bhakoo ON, Kaur I, Vaishnavi C (1988). Pityriasis (tinea) versicolor in infancy. Pediatr Dermatol.

[2] Abdollahimajd F, Niknezhad N, Niknejad N, Nikvar M (2019). Infantile hypopigmented pityriasis versicolor: two uncommon cases. Turk Pediatri Ars.

[3] Gupta AK, Batra R, Bluhm R, Boekhout T, Dawson TL Jr (2004). Skin diseases associated with Malassezia species. J Am Acad Dermatol.

[4] Camargo-Sánchez KA, Toledo-Bahena M, Mena-Cedillos C (2019). Pityriasis versicolor in children and adolescents: an update. Curr Fungal Infect Rep.

[5] Leung AK, Barankin B, Lam JM, Leong KF, Hon KL (2022). Tinea versicolor: an updated review. Drugs Context.

[6] Kallini JR, Riaz F, Khachemoune A (2014). Tinea versicolor in dark-skinned individuals. Int J Dermatol.

[7] Di Silverio A, Zeccara C, Serra F, Ubezio S, Mosca M (1995). Pityriasis versicolor in a newborn. Mycoses.

[8] Jubert E, Martín-Santiago A, Bernardino M, Bauzá A (2015). Neonatal pityriasis versicolor. Pediatr Infect Dis J.

[9] Ben Said Z, Boussofara L, Saidi W, Ghariani N, Denguezli M, Belajouza C, Nouira R (2010). Pityriasis versicolor in a 3-month-old boy. Arch Pediatr.

[10] Congly H (1984). Pityriasis versicolor in a 3-month-old boy. Can Med Assoc J.

